# Fluctuations in Serum Chloride and Acute Kidney Injury among Critically Ill Patients: A Retrospective Association Study

**DOI:** 10.3390/jcm8040447

**Published:** 2019-04-02

**Authors:** Tak Kyu Oh, In-Ae Song, Young-Tae Jeon, You Hwan Jo

**Affiliations:** 1 Department of Anesthesiology and Pain Medicine, Seoul National University Bundang Hospital, Seoul 13620, Korea; airohtak@hotmail.com (T.K.O.); ytjeon@snubh.org (Y.-T.J.); 2 Department of Emergency Medicine, Seoul National University Bundang Hospital, Seoul 13620, Korea; drakejo@snubh.org

**Keywords:** acute kidney injury, critical care, intensive care units

## Abstract

Exposure to dyschloremia among critically ill patients is associated with an increased risk of acute kidney injury (AKI). We aimed to investigate how fluctuations in serum chloride (Cl^−^) are associated with the development of AKI in critically ill patients. We retrospectively analyzed medical records of adult patients admitted to the intensive care unit (ICU) between January 2012 and December 2017. Positive and negative fluctuations in Cl^−^ were defined as the difference between the baseline Cl- and maximum Cl- levels and the difference between the baseline Cl^−^ and minimum Cl^−^ levels measured within 72 h after ICU admission, respectively. In total, 19,707 patients were included. The odds of developing AKI increased 1.06-fold for every 1 mmol L^−1^ increase in the positive fluctuations in Cl^−^ (odds ratio: 1.06; 95% confidence interval: 1.04 to 1.08; *p* < 0.001) and 1.04-fold for every 1 mmol L^−1^ increase in the negative fluctuations in Cl^−^ (odds ratio: 1.04; 95% confidence interval: 1.02 to 1.06; *p* < 0.001). Increases in both the positive and negative fluctuations in Cl- after ICU admission were associated with an increased risk of AKI. Furthermore, these associations differed based on the functional status of the kidneys at ICU admission or postoperative ICU admission.

## 1. Introduction

Acute kidney injury (AKI) is defined as an impairment of renal function [1], and is reported to occur in 2–18% of all inpatients, and 35.7–57% of all critically ill patients [2,3,4,5]. AKI that affects patients in intensive care units (ICUs) not only increases the duration of hospitalization and medical costs [6], but also increases in-hospital mortality [7]. Therefore, adequate prevention of AKI in ICUs is an important challenge in ICU patient management [8].

Serum chloride (Cl^−^) is the most common anion in the human body. Dyschloremia is a collective term for hypochloremia, in which the Cl^−^ level is below the normal range, and hyperchloremia, in which the Cl^−^ level is above the normal range [9]. Increased Cl^−^ levels induce hyperchloremic metabolic acidosis through physiologic compensation, whereas decreased Cl^−^ levels induce hypochloremic metabolic alkalosis. Both conditions are associated with increased risks of AKI [10,11]. It is important to understand the association between dyschloremia and AKI in the ICU because the Cl^−^ level can provide important information in the planning of a fluid management strategy [12].

It is well known that increases in Cl^−^ levels after ICU admission are associated with the development of AKI [13,14,15], while the association between a decrease in Cl^−^ levels and the development of AKI has not been extensively studied. Critically ill patients may experience a reduction in Cl^−^ levels after ICU admission due to the active loss of Cl^−^ in the gastrointestinal tract, impaired renal Cl^−^ reabsorption, hypotonic fluid infusion, excessive diuretics therapy, and malnutrition [16,17]. These conditions may be associated with the development of AKI. Thus, when studying the association between Cl^−^ levels and the incidence of AKI among critically ill patients, fluctuations of Cl^−^ levels (increases and decreases) must be considered.

Therefore, this study aimed to investigate the association between the total, positive, and negative fluctuations in Cl^−^ levels and the incidence of AKI.

## 2. Materials and Methods

### 2.1. Study Design and Subjects

This retrospective observational study was approved by the Institutional Review Board (IRB) of Seoul National University Bundang Hospital (IRB approval number B-1806/474-105). The IRB exempted the need for informed consent, considering the retrospective study design. The medical records of adult patients aged ≥18 years admitted to the ICU between January 2012 and December 2017 were analyzed. For single patients admitted to the ICU twice or more during the study period, only the last ICU admission in which the patient could be in the most critical condition was included in the analysis. Patients whose medical records, regarding Cl^−^ and creatinine, were incomplete or missing were excluded from the analysis. Patients with an estimated glomerular filtration rate (eGFR) <15 mL min^−1^ 1.73 m^−2^, patients with end-stage renal disease (ESRD) who underwent chronic renal replacement therapy (RRT) before ICU admission, and patients with undiagnosed AKI before ICU admission were also excluded.

This study succeeds a previous study [18] that analyzed the medical records of patients in the surgical ICU at our institution from 2011 to 2016. The previous study reported that exposure to hyperchloremia in the postoperative period in the surgical ICU was not associated with the incidence of AKI. This study differs from the previous study that analyzed the positive or negative fluctuations in Cl^−^ within 72 h after ICU admission; previous studies have analyzed the increases in the preoperative Cl^−^ to the maximum Cl^−^ in 0–3 days postoperatively.

### 2.2. Fluctuations in Cl- Levels (Independent Variables)

For the purpose of this study, the Cl- level on ICU admission (baseline Cl-) was defined as that measured within 24 h after ICU admission, and the Cl- level closest to the ICU admission time. Positive fluctuations in Cl- were defined as the difference between the baseline Cl- and the maximum Cl- levels measured within 72 h after ICU admission, while negative fluctuations in Cl- were defined as the difference between the baseline Cl- and the minimum Cl- levels measured within 72 h after ICU admission. Lastly, the total fluctuations in Cl- were defined as the difference between the minimum and maximum Cl- levels measured within 72 h after ICU admission. For example, if baseline Cl-, maximum Cl-, and minimum Cl- levels were 107 mmol L^−1^, 111 mmol L^−1^, and 105 mmol L^−1^, respectively, the total positive and negative fluctuations were 6 mmol L^−1^ (111–105 mmol L^−1^), 4 mmol L^−1^ (111–107 mmol L^−1^), and 2 mmol L^−1^ (107–105 mmol L^−1^), respectively. In situations where no maximum or minimum value of Cl- within 72 h after ICU admission was noted, the positive or negative fluctuation of Cl-, respectively, was considered as 0. In those cases, the total fluctuation was calculated using baseline Cl- level. For example, if baseline Cl-, maximum Cl-, and minimum Cl- levels were 105 mmol L^−1^, 111 mmol L^−1^, and 107 mmol L^−1^, respectively, the total, positive, and negative fluctuations were 6 mmol L^−1^ (111–105 mmol L^−1^), 6 mmol L^−1^ (111–105 mmol L^−1^), and 0 mmol L^−1^ (no minimum Cl- level), respectively.

### 2.3. Potential Covariates

Data regarding demographics (sex, age, and body mass index), Acute Physiology, Chronic Health Evaluation II, comorbidities at ICU admission (eGFR, mL min^−1^ 1.73 m^−2^, hypertension, diabetes mellitus, history of ischemic heart disease and cerebrovascular disease, chronic obstructive lung disease, liver disease (liver cirrhosis, hepatitis, and fatty liver), anemia (hemoglobin <10 g dL^−1^), cancer status regarding hospital admission through the emergency department, postoperative admission status, and the admission department (internal medicine/neurologic center/postcardiothoracic surgery/post-other surgery) at the time of ICU admission were collected. Information regarding fluid administration (i.e., NaCl 0.9%, balanced crystalloid, and hydroxyethyl starch (all in mL)) for 72 h after ICU admission was collected. Additionally, the maximum value of the cystatin c level (mg dL^−1^) for 72 h after ICU admission was collected. Finally, the number of Cl- level measurements taken for 72 h after ICU admission were collected. The Modification of Diet in Renal Disease equation was used to calculate the eGFR before ICU admission [19]: eGFR (mL min^−1^ 1.73 m^−2^) = 186 × (Creatinine)^−1.154^ × (Age)^−0.203^ × (0.742 if female).

### 2.4. Acute Kidney Injury within 72 h after ICU Admission (Dependent Variable)

The Kidney Disease: Improving Global Outcomes (KDIGO) criteria and grading method were used to diagnose AKI (Appendix A) [20]. Considering the differences in the duration of urinary catheterization among the patients, only the serum creatinine (mg dL^−1^) level was used to diagnose AKI. The serum creatinine value measured within 1 month before ICU admission closest to the time of ICU admission was used as the baseline creatinine concentration for AKI diagnosis. The serum creatinine level measured within 72 h after ICU admission was used to diagnose AKI.

### 2.5. Endpoint

This study investigated the associations between total, positive, and negative fluctuations in Cl- within 72 h after ICU admission and the total incidence of AKI and AKI stage ≥2. In addition, we investigated relationships between total, positive, and negative fluctuations in serum Cl^−^ with the maximum serum cystatin C level for 72 h after ICU admission.

### 2.6. Statistical Analysis

The patients’ baseline characteristics were expressed as means and standard deviations (SDs) or numbers and proportions. The log odds of AKI occurrence and fluctuations in Cl^−^ were presented as restricted cubic splines (RCSs). After confirming a linear relationship between the fluctuation in Cl^−^ and log odds of developing AKI in RCSs, the fluctuation in Cl^−^ was included in the logistic regression model as a continuous variable. A univariable logistic regression analysis was performed to investigate the association of each covariate with the incidence of the dependent variable (AKI). Covariates with *p* < 0.1 were selected from the univariable logistic regression model, and were controlled for in the final multivariable logistic regression analysis. In the multivariable logistic regression analysis, total fluctuations in Cl^−^ were included in another multivariable logistic regression model with positive and negative fluctuations in Cl^−^ to avoid multicollinearity within variables.

Next, considering that baseline kidney function is a major risk factor of AKI [21], the interaction between fluctuations in Cl^−^ and eGFR and the incidence of AKI before ICU admission were investigated. After confirming that there was a significant interaction between fluctuations in Cl^−^ and eGFR with the incidence of AKI, we performed a subgroup analysis with four eGFR groups (eGFR ≥90, <90, <60, and <30 mL min^−1^ 1.73 m^−2^). Lastly, the interaction between fluctuations in Cl^−^ and postoperative ICU admission for the incidence of AKI were investigated, and the significant interactions were also confirmed. Therefore, we performed a subgroup analysis based on postoperative ICU admission. To reduce type I errors due to multiple comparisons in the subgroup analysis, the Bonferroni correction was used [22]. The same method was used in the analysis of stage ≥2 as a dependent variable. The results of the logistic regression analysis were expressed as odds ratios (ORs) and 95% confidence intervals (CIs). Additionally, considering that the serum cystatin C was a marker of renal function in the detection of early AKI [23], we performed a generalized linear regression analysis to investigate the association between fluctuation in serum Cl^−^ and the maximum serum cystatin C level for 72 h after ICU admission. In this generalized linear model (GLM), gamma distribution and the log link function were assumed for the dependent variable (maximum cystatin C level within 72 h after ICU admission). All covariates were included in the GLM. The results of GLM were expressed as the exponentiated (exp) regression coefficient (coef) with 95% CIs. All analyses were performed using SPSS version 24.0 (IBM Corp., Armonk, NY) and R program (version 3.5.2 with R packages), with the level of statistical significance set at *p* < 0.05.

## 3. Results

**Figure 1:**
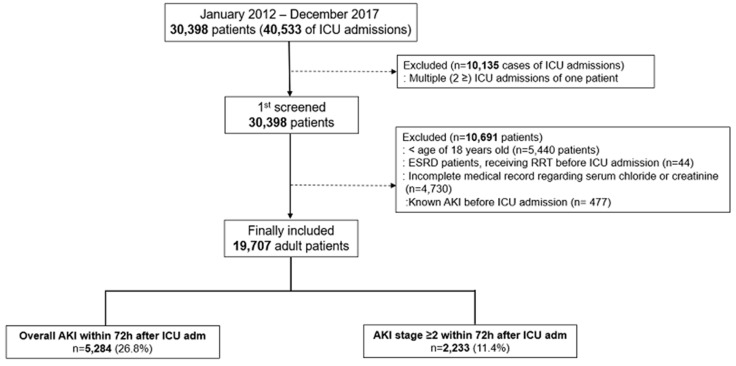
Flow chart of patient selection.

There was a total of 40,533 ICU admissions between 2012 and 2017. Of these, 10,135 admission cases in which a single patient was admitted twice or more were excluded. Next, 5440 patients younger than 17 years, 44 ESRD patients who received RRT before ICU admission, 4730 patients with incomplete medical records regarding serum Cl- or creatinine levels, and 477 patients with undiagnosed AKI before admission were excluded, and the remaining 19,707 patients were finally included. There was a total of 5284 (26.8%) AKI cases within 72 h after ICU admission; 2233 (11.4%) patients had AKI stage ≥2 (Figure 1). Table 1 shows the baseline characteristics of these patients. The mean (SD) values of the total, positive, and negative fluctuations in Cl- were 7.0 (5.7), 4.4 (4.1), and 2.9 (4.6), respectively.

### 3.1. AKI within 72 h after ICU Admission Based on Cl- Fluctuations

**Figure 2:**
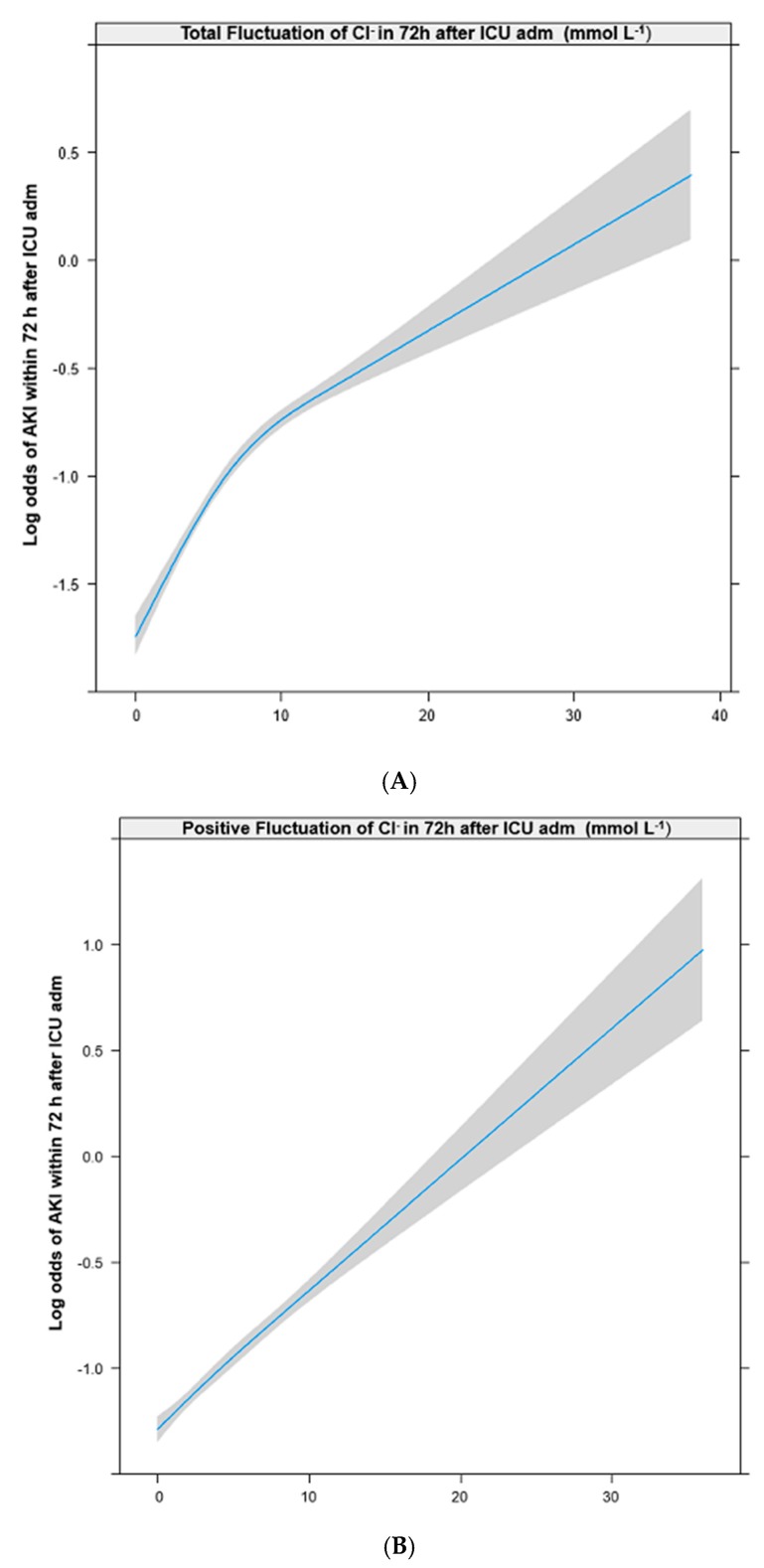

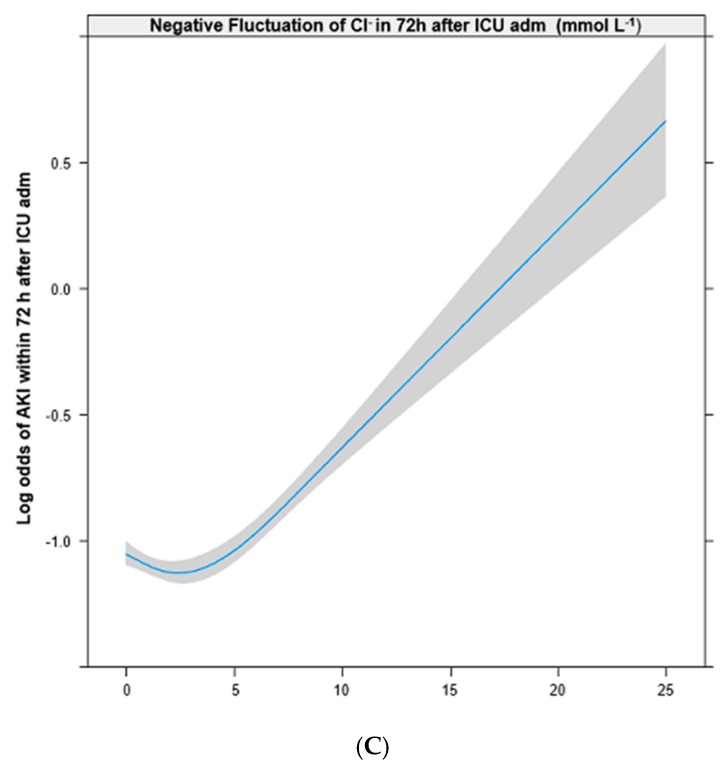
Restricted cubic spline between total (**A**), positive (**B**), and negative (**C**) fluctuations in serum chloride within 72 h after ICU admission and occurrence of AKI. ICU, intensive care unit; AKI, acute kidney injury. RRT, renal replacement therapy.

The RCSs in Figure 2 show that the log odds of developing AKI had positive and linear relationships with total (A), positive (B), and negative fluctuations (C) in Cl^−^ levels. Appendix B shows the results of the univariable logistic regression analysis of the associations between the individual covariates and AKI. Table 2 shows the results of the multivariable logistic regression analysis adjusted for the covariates selected from the univariable logistic regression analysis. The odds of developing AKI increased 1.05-fold for every 1 mmol L^−1^ increase in the total fluctuations in Cl^−^ (OR: 1.05; 95% CI: 1.03 to 1.06; *p* < 0.001), 1.06-fold for every 1 mmol L^−1^ increase in the positive fluctuations in Cl^−^ (OR: 1.06; 95% CI: 1.04 to 1.08; *p* < 0.001), and 1.04-fold for every 1 mmol L^−1^ increase in the negative fluctuations in Cl^−^ (OR: 1.04; 95% CI: 1.02 to 1.06; *p* < 0.001). The results of the subgroup analysis for total AKI based on the preadmission eGFR status and the postoperative ICU admission status are shown in Table 3 and Table 4, respectively.

### 3.2. AKI Stage ≥2 within 72 h after ICU Admission According to Cl^−^ Fluctuation

Appendix B shows the results of the univariable logistic regression analysis of the associations between the individual covariates and AKI stage ≥2. Table 2 shows the results of the multivariable logistic regression analysis adjusted for the covariates selected from the univariable logistic regression analysis. The odds of developing stage ≥2 AKI increased 1.08-fold for every 1 mmol L^−1^ increase in the total fluctuations in Cl^−^ (OR: 1.08; 95% CI: 1.06 to 1.10; *p* < 0.001), 1.09-fold for every 1 mmol L^−1^ increase in the positive fluctuations in Cl^−^ (OR: 1.09; 95% CI: 1.07 to 1.11; *p* < 0.001), and 1.09-fold for every 1 mmol L^−1^ increase in the negative fluctuations in Cl^−^ (OR: 1.09; 95% CI: 1.06 to 1.11; *p* < 0.001). The results of the subgroup analysis for AKI stage ≥2 according to preadmission eGFR grouping and postoperative ICU admission status are shown in Table 3 and Table 4, respectively.

### 3.3. Fluctuation in Cl^−^ and Maximum Serum Cystatin Level during the 72 h after ICU Admission

Serum cystatin C was measured in the 2021 patients at least once within 72 h after ICU admission. In these patients, generalized linear regression analysis was performed, and the results of the GLM are presented in Table 5. A 1 mmol L^−1^ increase in the negative fluctuation in Cl^−^ was associated with a 1.4% increase of maximum cystatin C level (exp coef: 0.014, 95% CI: 0.002 to 0.026; *p* = 0.026), while total fluctuation of Cl^−^ (*p* = 0.374) and positive fluctuation of Cl^−^ (0.682) were not associated with the maximum cystatin C level.

## 4. Discussion

This study showed that both positive and negative fluctuations in Cl^−^ within 72 h after ICU admission were significantly associated with the potential risk of AKI in a mixed ICU adult population. This association was also observed for AKI stage ≥2. In the subgroup analysis based on preadmission eGFR grouping, the association between positive fluctuations in Cl^−^ and AKI was more evident in the eGFR ≥90 mL min^−1^ 1.73 m^−2^ group, while the association between negative fluctuations in Cl^−^ and AKI was more evident in the eGFR <90 or <60 mL min^−1^ 1.73 m^−2^ group. Additionally, both positive and negative fluctuations in Cl^−^ were associated with the risk of AKI in patients without postoperative ICU admission, while only positive fluctuations in Cl^−^ were significantly associated with the risk of AKI in patients with postoperative admissions.

The most novel finding of this study is that we reported that the negative fluctuations in Cl^−^ could also be associated with the risk of AKI in critically ill patients. While the association between positive fluctuations in Cl^−^ and AKI were reported in previous studies [13,14,15], the association regarding the negative fluctuations has yet to be reported. While positive fluctuations in Cl^−^ could be caused by fluid resuscitation [24], negative fluctuations in Cl^−^ could be caused by a loss of active Cl^−^ from the gastrointestinal tract, impaired renal Cl^−^ reabsorption, and an infusion of hypotonic fluid [16,17], which might be related to AKI [25]. Additionally, hypochloremia might be caused by negative fluctuations in Cl^−^, which is a common and independent poor prognostic factor in critically ill patients [26]. Although our findings regarding positive fluctuations in CI^−^ were consistent with those of a meta-analysis published in 2015 [27], there was another meta-analysis, published in 2018, which concluded that the relationship between the use of chloride-rich solution and AKI remains controversial [28]. Therefore, future studies should investigate the effect of positive or negative fluctuations in Cl^−^ on AKI.

Another interesting finding was that the interactions related to AKI existed between the eGFR status at the time of ICU admission and total fluctuations in Cl^−^. The results of the subgroup analysis based on eGFR grouping showed that the positive fluctuations in Cl^−^ tended to be more frequently associated with AKI in patients with normal kidney function (eGFR ≥90 mL min^−1^ 1.73 m^−2^). In contrast, negative fluctuations in Cl^−^ tended to be more frequently associated with AKI in patients with CKD (eGFR <90 or 60 mL min^−1^ 1.73 m^−2^). There are several potential explanations for our findings. First, patients with normal kidney function at ICU admission might have received more chloride-rich fluid resuscitation than CKD patients; this might have impacted the positive fluctuations in Cl^−^. Secondly, since CKD patients often had disruptions in their acid–base balance [25], the negative fluctuations in Cl^−^ might have had a greater impact on the patients with CKD. Lastly, the impact of both positive and negative fluctuations in Cl^−^ was not significant in patients with CKD 4 or 5 (<30). There is a possibility that the fluctuations in Cl^−^ were minimized by physicians for such severe CKD patients, thus impacting these results in patients with CKD 4 or 5.

The difference in the results regarding positive fluctuations in Cl^−^ between this study and our previous study is also interesting [18]. In our previous study, we found that hyperchloremia (>110 mmol L^−1^) was not associated with postoperative AKI in the surgical ICU, and there was a positive association in the increase from the preoperative Cl^−^ (which was measured within 1 month prior to surgery) to the maximum Cl^−^ measured 0–3 days postoperatively in patients with a CKD stage ≥3. The differences between two studies might be caused by the study designs. Our previous study might have been affected by fluid resuscitation or blood loss during surgery, while the present study was not affected by these factors. In general, more fluid administration is required to replace ongoing bleeding or insensible loss of fluid during surgery [29], so that the impact of the Cl^−^ load on AKI would be different from that in the ICU.

Although the serum cystatin C level was measured in only 2021 patients (10.2%) for 72 h after ICU admission in this study, our results regarding the relationship between Cl^−^ fluctuation and cystatin level were also notable. In this study, only the negative Cl^−^ fluctuation was associated with an increase in serum cystatin level during the 72 h after ICU admission. Considering that cystatin C is known as a marker of renal function in AKI [23], our results suggest that a decrease of Cl^−^ level might be an associated factor for development of AKI after ICU admission. In addition, the relationship between AKI and the increase of Cl^−^ could be caused by fluid administration. Furthermore, the significant relationship between the positive fluctuation and development of AKI could be related to clinical situations that require fluid administration. However, a decrease of Cl^−^ was more related to kidney damage via hypochloremic metabolic alkalosis [10] than to an increase in Cl^−^. Considering the relatively small sample size of patients who had their serum cystatin C measured for 72 h after ICU admission in this study, more studies should be performed in the future to confirm the relationship between dyschloremia, cystatin C levels, and AKI.

This study had a number of limitations. First, due to the retrospective cohort design, selection bias may have occurred during the data collection process. To minimize this bias, all data were collected by a medical record technician blinded from the purpose of this study. Second, this study was performed at a single center, and therefore its results may have limited generalizability. Third, Cl^−^ levels were not measured during the same period, using the same method for all patients included in this study. Fourth, since patients who developed AKI were much more likely to develop dyschloremia due to the inability of their kidneys to effectively regulate Cl^−^ levels, there is a possibility that AKI may precede changes in Cl^−^, and thus might confound our study conclusions. Fifth, we could only use serum creatinine concentrations for the accurate diagnosis of AKI in accordance with the KDIGO criteria due to a lack of accurate urine output data. The exclusion of urine output data may reduce the accuracy and sensitivity of AKI diagnosis, especially for the diagnosis of more severe stages of AKI (stage 2 or 3) [30,31]. Lastly, in this study, we did not evaluate various biomarkers for AKI such as beta-2 microglobulin, liver-type fatty acid binding protein, and neutrophil gelatinase-associated lipocalin. Considering there are many biomarkers for the early detection of AKI [32], more biomarkers are needed to evaluate the direct effect of Cl^−^ fluctuation on AKI development.

## 5. Conclusions

This study showed that an increase in both the positive and negative fluctuations in Cl- after ICU admission were associated with an increased risk of AKI after ICU admission. Furthermore, these associations differed based on the kidney functionality at ICU admission or postoperative ICU admission. However, the results should be interpreted carefully considering the retrospective design, and future studies should be performed using biomarkers for AKI.

## Figures and Tables

**Table 1:** Baseline characteristics of adult patients admitted to the ICU between 2012 and 2017.

Variable		Total (19,707)	Mean	SD
Sex: male		11,412 (57.9%)		
Age, year			63.8	15.9
Body mass index, kg m^−2^			23.6	3.9
Comorbidities at ICU admission				
	APACHE II		20.3	10.0
	eGFR ^a^: ≥90	12,164 (61.7%)		
	60–90	4079 (20.7%)		
	30–60	2163 (11.0%)		
	<30	1301 (6.6%)		
	Hypertension	8511 (43.2%)		
	Diabetes mellitus	1775 (9.0%)		
	Ischemic heart disease	481 (2.4%)		
	Cerebrovascular disease	886 (4.5%)		
	Chronic obstructive lung disease	868 (4.4%)		
	Liver disease (LC, hepatitis, fatty liver)	649 (3.3%)		
	Anemia (Hb <10 g dL^−1^)	7266 (36.9%)		
	Cancer	4137 (21.0%)		
Characteristics of ICU admission				
	Admission through emergency department	11,435 (58.0%)		
	Postoperative admission	8728 (44.3%)		
	Admission department			
	Internal medicine	4231 (21.5%)		
	Neurologic center	4805 (24.4%)		
	Cardiothoracic surgical department	6093 (30.9%)		
	Other surgical departments	4578 (23.2%)		
	Length of ICU stay, day		3.2	10.4
	Length of hospital stay, day		13.3	20.5
Fluid administration for 72 h after ICU admission				
	NaCl 0.9%, mL		1745.5	2124.1
	Balanced crystalloid, mL		505.5	862.5
	Hydroxyethyl starch, mL		79.4	270.1
	Transfusion of packed RBC	8530 (43.3%)		
Serum chloride (Cl^−^) in ICU, mmol L^−1^				
	Cl^−^ on ICU admission		106.4	6.2
	The number of measurements for 72 h after ICU admission		3.2	1.0
	Total fluctuation of Cl^−^ for 72 h after ICU admission ^b^		7.0	5.7
	Positive fluctuation of Cl^−^ for 72 h after ICU admission ^c^		4.4	4.1
	Negative fluctuation of Cl^−^ for 72 h after ICU admission ^d^		2.9	4.6
Max cystatin C level mg dL^−1^ for 72 h after ICU adm (*n =* 2,021)			2.0	1.2
Total AKI within 72 h after ICU admission		5284 (26.8%)		
AKI stage ≥2 within 72 h after ICU admission		2233 (11.4%)		
RRT after ICU admission (within 72 h)		468 (2.4%)		

^a^ eGFR (mL min ^−1^ 1.73 m^−2^): 186 × (Creatinine)^−1.154^ × (Age)^−0.203^ × (0.742 if female). ^b^ Total fluctuation of Cl^−^: (Maximum Cl^−^– Minimum Cl^−^) for 72 h after ICU admission. ^c^ Positive fluctuation of Cl^−^: (Maximum Cl^−^ – Preadmission Cl^−^) for 72 h after ICU admission. ^d^ Negative fluctuation of Cl^−^: (Preadmission Cl^−^ – Minimum Cl^−^) for 72 h after ICU admission. ICU, intensive care unit; APACHE, acute physiology and chronic health evaluation; eGFR, estimated glomerular filtration rate; LC, liver cirrhosis; Hb, hemoglobin; RBC, red blood cell; Max, maximum; AKI, acute kidney injury; RRT, renal replacement therapy.

**Table 2:** Multivariable logistic regression analysis for total AKI and AKI stage ≥2 after ICU admission according to fluctuations of serum chloride (mmol L^−1^).

Variables	Odds Ratio (95% CI)	*p*-Value
**Dependent variables: Total AKI**		
Total fluctuation of Cl^− a^ (model 1)	**1.05** (1.03, 1.06)	**<0.001**
Interaction: Total fluctuation of Cl^− a,^* eGFR ^b^ ≥90	1	(<0.001)
Total fluctuation of Cl^− a,^* eGFR ^b^: 60–90	1.02 (1.00, 1.04)	0.114
Total fluctuation of Cl^− a,^* eGFR ^b^: 30–60	1.02 (1.00, 1.05)	0.153
Total fluctuation of Cl^− a,^* eGFR ^b^: <30	0.90 (0.88, 0.93)	<0.001
Interaction: Total fluctuation of Cl^− a,^* postoperative admission	0.96 (0.95, 0.98)	<0.001
Positive fluctuation of Cl^− c^ (model 2)	**1.06** (1.04, 1.08)	**<0.001**
Interaction: Positive fluctuation of Cl^− a,^*eGFR ^b^ ≥90	1	(<0.001)
Positive fluctuation of Cl^− a,^* eGFR ^b^:60–90	1.02 (0.99, 1.04)	0.259
Positive fluctuation of Cl^− a,^* eGFR ^b^:30–60	1.00 (0.97, 1.03)	0.812
Positive fluctuation of Cl^− a,^* eGFR ^b^:<30	0.87 (0.84, 0.90)	<0.001
Interaction: Positive fluctuation of Cl^− a,^* postoperative admission	0.98 (0.96, 1.00)	0.095
Negative fluctuation of Cl^− c^ (model 2)	**1.04** (1.02, 1.06)	**<0.001**
Interaction: Negative fluctuation of Cl^− a,^*eGFR ^b^ ≥90	1	(<0.001)
Negative fluctuation of Cl^− a,^* eGFR ^b^: 60–90	1.02 (0.99, 1.06)	0.145
Negative fluctuation of Cl^− a,^* eGFR ^b^: 30–60	1.05 (1.01, 1.09)	0.006
Negative fluctuation of Cl^− a,^* eGFR ^b^: <30	0.94 (0.91, 0.98)	0.003
Interaction: Negative fluctuation of Cl^− a,^* postoperative admission	0.94 (0.92, 0.97)	<0.001
**Dependent variables: AKI stage ≥2**		
Total fluctuation of Cl^− a^	**1.08** (1.06, 1.10)	**<0.001**
Interaction: Total fluctuation of Cl^− a,^* eGFR ^b^: ≥90	1	(<0.001)
Total fluctuation of Cl^− a,^* eGFR ^b^: 60–90	1.00 (0.97, 1.03)	0.853
Total fluctuation of Cl^− a,^* eGFR ^b^: 30–60	0.96 (0.92, 0.99)	0.022
Total fluctuation of Cl^− a,^* eGFR ^b^: <30	0.89 (0.86, 0.92)	<0.001
Interaction: Total fluctuation of Cl^− a,^* postoperative admission	0.96 (0.93, 0.98)	<0.001
Positive fluctuation of Cl^− c^	**1.09** (1.07, 1.11)	**<0.001**
Interaction: Positive fluctuation of Cl^− a,^*eGFR ^b^ ≥90	1	(<0.001)
Positive fluctuation of Cl^− a,^* eGFR ^b^: 60–90	1.00 (0.96, 1.03)	0.881
Positive fluctuation of Cl^− a,^* eGFR ^b^: 30–60	0.92 (0.88, 0.96)	<0.001
Positive fluctuation of Cl^− a,^* eGFR ^b^: <30	0.87 (0.84, 0.91)	<0.001
Interaction: Positive fluctuation of Cl^− a,^* postoperative admission		
Negative fluctuation of Cl^− d^	**1.09** (1.06, 1.11)	**<0.001**
Interaction: Negative fluctuation of Cl^− a,^*eGFR ^b^ ≥90	1	(<0.001)
Negative fluctuation of Cl^− a,^* eGFR ^b^: 60–90	0.99 (0.95, 1.04)	0.789
Negative fluctuation of Cl^− a,^* eGFR ^b^: 30–60	1.00 (0.95, 1.04)	0.853
Negative fluctuation of Cl^− a,^* eGFR ^b^: <30	0.91 (0.87, 0.95)	<0.001
Interaction: Negative fluctuation of Cl^− a,^* postoperative admission	0.91 (0.89, 0.94)	<0.001

Covariates of *p* < 0.1 in univariable logistic regression analysis (Appendix B) were included to adjust the multivariable logistic regression model. ^a^ Total fluctuation of Cl^−^ (mmol L^−1^): (Maximum Cl^−^– Minimum Cl^−^) for 72 h after ICU admission. ^b^ eGFR (mL min ^−1^ 1.73 m^−2^): 186 × (Creatinine)^−1.154^ × (Age)^−0.203^ × (0.742 if female). ^c^ Positive fluctuation of Cl^−^ (mmol L^−1^): (Maximum Cl^−^ – Preadmission Cl^−^) for 72 h after ICU admission. ^d^ Negative fluctuation of Cl^−^ (mmol L^−1^): (Preadmission Cl^−^ – Minimum Cl^−^) for 72 h after ICU admission. AKI, acute kidney injury; ICU, intensive care unit; eGFR, estimated glomerular filtration rate.

**Table 3:** Multivariable logistic regression analysis for total AKI and AKI stage ≥2 after ICU admission according to preadmission eGFR ^a^ group.

Variables	Odds Ratio (95% CI)	*p* *
**Dependent variable: Total AKI**		
eGFR ^a^ ≥90 (*n =* 12,164)		
Total fluctuation of Cl^− b^ (per 1 mmol L^−1^)	**1.04** (1.02, 1.05)	**<0.001**
Positive fluctuation of Cl^− c^ (per 1 mmol L^−1^)	**1.05** (1.03, 1.06)	**<0.001**
Negative fluctuation of Cl^− d^ (per 1 mmol L^−1^)	1.01 (0.99, 1.03)	0.204
eGFR ^a^ <90 (*n =* 7,543)		
Total fluctuation of Cl^− b^ (per 1 mmol L^−1^)	**1.02** (1.01, 1.04)	**0.002**
Positive fluctuation of Cl^− c^ (per 1 mmol L^−1^)	**1.02** (1.00, 1.04)	**0.024**
Negative fluctuation of Cl^− d^ (per 1 mmol L^−1^)	**1.03** (1.01, 1.05)	**0.004**
eGFR ^a^: <60 (*n =* 3,464)		
Total fluctuation of Cl^− b^ (per 1 mmol L^−1^)	1.01 (0.99, 1.03)	0.239
Positive fluctuation of Cl^− c^ (per 1 mmol L^−1^)	0.99 (0.97, 1.01)	0.529
Negative fluctuation of Cl^− d^ (per 1 mmol L^−1^)	**1.03** (1.01, 1.06)	**0.004**
eGFR ^a^: <30 (*n =* 1,301)		
Total fluctuation of Cl^− b^ (per 1 mmol L^−1^)	0.98 (0.95, 1.01)	0.150
Positive fluctuation of Cl^− c^ (per 1 mmol L^−1^)	0.99 (0.91, 1.01)	0.052
Negative fluctuation of Cl^− d^ (per 1 mmol L^−1^)	1.01 (0.98, 1.05)	0.469
**Dependent variables: AKI stage ≥2**		
eGFR ^a^ ≥90 (*n =* 12,164)		
Total fluctuation of Cl^− b^ (per 1 mmol L^−1^)	**1.06** (1.04, 1.08)	**<0.001**
Positive fluctuation of Cl^− c^ (per 1 mmol L^−1^)	**1.07** (1.05, 1.09)	**<0.001**
Negative fluctuation of Cl^− d^ (per 1 mmol L^−1^)	**1.04** (1.02, 1.06)	**0.001**
eGFR ^a^ <90 (*n =* 7,543)		
Total fluctuation of Cl^− b^ (per 1 mmol L^−1^)	**1.03** (1.01, 1.05)	**0.002**
Positive fluctuation of Cl^− c^ (per 1 mmol L^−1^)	1.02 (1.00, 1.04)	0.059
Negative fluctuation of Cl^− d^ (per 1 mmol L^−1^)	**1.04** (1.02, 1.06)	**0.001**
eGFR ^a^: <60 (*n =* 3,464)		
Total fluctuation of Cl^− b^ (per 1 mmol L^−1^)	1.01 (0.99, 1.03)	0.317
Positive fluctuation of Cl^− c^ (per 1 mmol L^−1^)	0.99 (0.96, 1.01)	0.280
Negative fluctuation of Cl^− d^ (per 1 mmol L^−1^)	**1.04** (1.01, 1.07)	**0.003**
eGFR ^a^: <30 (*n =* 1,301)		
Total fluctuation of Cl^− b^ (per 1 mmol L^−1^)	1.00 (0.97, 1.04)	0.788
Positive fluctuation of Cl^− c^ (per 1 mmol L^−1^)	0.99 (0.95, 1.03)	0.489
Negative fluctuation of Cl^− d^ (per 1 mmol L^−1^)	1.02 (0.98, 1.06)	0.247

* p < 0.013 was considered as statistically significant after Bonferroni correction. Covariates of *p* < 0.1 in univariable logistic regression analysis (Appendix B) were included to adjust the multivariable logistic regression model. ^a^ eGFR (mL min ^−1^ 1.73m^−2^): 186 × (Creatinine)^−1.154^ × (Age)^−0.203^ × (0.742 if female). ^b^ Total fluctuation of Cl^−^ (mmol L^−1^): (Maximum Cl^−^ – Minimum Cl^−^) for 72 h after ICU admission. ^c^ Positive fluctuation of Cl^−^ (mmol L^−1^): (Maximum Cl^−^ – Preadmission Cl^−^) for 72 h after ICU admission. ^d^ Negative fluctuation of Cl^−^ (mmol L^−1^): (Preadmission Cl^−^ – Minimum Cl^−^) for 72 h after ICU admission.

**Table 4:** Multivariable logistic regression analysis for total AKI and AKI (stage ≥2) after ICU admission according to postoperative admission.

Variables	Odds ratio (95% CI)	*p* *
**Dependent variable: Total AKI**		
Postoperative admission (*n =* 8,728)		
Total fluctuation of Cl^− a^ (per 1 mmol L^−1^)	1.01 (1.00, 1.02)	0.170
Positive fluctuation of Cl^− b^ (per 1 mmol L^−1^)	**1.03** (1.01, 1.04)	**0.002**
Negative fluctuation of Cl^− c^ (per 1 mmol L^−1^)	0.99 (0.97, 1.01)	0.254
Non-postoperative admission (*n =* 10,992)		
Total fluctuation of Cl^− a^ (per 1 mmol L^−1^)	**1.04** (1.02, 1.05)	**<0.001**
Positive fluctuation of Cl^− b^ (per 1 mmol L^−1^)	**1.04** (1.02, 1.05)	**<0.001**
Negative fluctuation of Cl^− c^ (per 1 mmol L^−1^)	**1.04** (1.02, 1.05)	**<0.001**
**Dependent variables: AKI stage ≥2**		
Postoperative admission (*n =* 8,728)		
Total fluctuation of Cl^− a^ (per 1 mmol L^−1^)	1.01 (0.99, 1.03)	0.300
Positive fluctuation of Cl^− b^ (per 1 mmol L^−1^)	**1.03** (1.00, 1.05)	**0.023**
Negative fluctuation of Cl^− c^ (per 1 mmol L^−1^)	0.99 (0.96, 1.02)	0.396
Non-postoperative admission (*n =* 10,992)		
Total fluctuation of Cl^− a^ (per 1 mmol L^−1^)	**1.05** (1.04, 1.07)	**<0.001**
Positive fluctuation of Cl^− b^ (per 1 mmol L^−1^)	**1.05** (1.03, 1.07)	**<0.001**
Negative fluctuation of Cl^− c^ (per 1 mmol L^−1^)	**1.06** (1.04, 1.08)	**<0.001**

* p < 0.025 was considered as statistically significant after Bonferroni correction. Covariates of *p* < 0.1 in univariable logistic regression analysis (Appendix B) were included to adjust the multivariable logistic regression model. ^a^ Total fluctuation of Cl^−^ (mmol L^−1^): (Maximum Cl^−^ – Minimum Cl^−^) for 72 h after ICU admission. ^b^ Positive fluctuation of Cl^−^ (mmol L^−1^): (Maximum Cl^−^ – Preadmission Cl^−^) for 72 h after ICU admission. ^c^ Negative fluctuation of Cl^−^ (mmol L^−1^): (Preadmission Cl^−^ – Minimum Cl^−^) for 72 h after ICU admission. AKI, acute kidney injury; ICU, intensive care unit; eGFR, estimated glomerular filtration rate.

**Table 5:** Generalized linear regression model for maximum cystatin C level within 72 h after ICU admission according to fluctuation of Cl^−^ (*n =* 2,021).

Variables	Exp Coef (95% CI)	p *
Dependent variable: maximum cystatin C level (mmol L^−1^)		
Total fluctuation of Cl^− a^ (per 1 mmol L^−1^, model 1)	0.004 (−0.005, 0.013)	0.374
Positive fluctuation of Cl^− b^ (per 1 mmol L^−1^, model 2)	−0.002 (−0.012, 0.008)	0.682
Negative fluctuation of Cl^− c^ (per 1 mmol L^−1^, model 2)	**0.014** (0.002, 0.026)	0.026

* In the generalized linear model, gamma distribution and the log link function were assumed for the dependent variable (maximum cystatin C level within 72 h after ICU admission). All covariates were included in the model. ^a^ Total fluctuation of Cl^−^ (mmol L^−1^): (maximum Cl^−^ – minimum Cl^−^) for 72 h after ICU admission. ^b^ Positive fluctuation of Cl^−^ (mmol L^−1^): (maximum Cl^−^ – preadmission Cl^−^) for 72 h after ICU admission. ^c^ Negative fluctuation of Cl^−^ (mmol L^−1^): (preadmission Cl^−^ – minimum Cl^−^) for 72 h after ICU admission. Exp, exponentiated; Coef, coefficient; APACHE, acute physiology and chronic health evaluation; eGFR, estimated glomerular filtration rate.

## Appendix A

**Table A1:** Staging of postoperative acute kidney injury (KDIGO).

Stage	Serum Creatinine
1	1.5–1.9 times baseline or ≥0.3 mg dL^−1^ increase within 72 h after ICU admission
2	2.0–2.9 times baseline within 72 h after ICU admission
3	3.0 times baseline or increase in serum creatinine to ≥4.0 mg dL^−1^ or initiation of RRT within 72 h after ICU admission

KDIGO, kidney disease: improving global outcomes; RRT, renal replacement therapy.

## Appendix B

**Table A2:** Univariable logistic regression analysis of covariates for occurrence of total AKI and AKI stage ≥2 during 72 h after ICU admission.

Variables			Total AKI		AKI Stage ≥2	
			OR (95% CI)	*p*-Value	OR (95% CI)	*p*-Value
Sex: male			1.09 (1.02–1.16)	0.012	1.08 (0.99–1.18)	0.093
Age, year			1.02 (1.02–1.02)	<0.001	1.01 (1.01–1.02)	<0.001
Body mass index, kg m^−2^			0.96 (0.95–0.97)	<0.001	0.93 (0.92–0.95)	<0.001
APACHE II			1.04 (1.04–1.04)	<0.001	1.04 (1.03–1.04)	<0.001
Comorbidities at ICU admission						
	Hypertension		1.30 (1.22–1.38)	<0.001	1.16 (1.06–1.26)	0.001
	Diabetes mellitus		1.51 (1.36–1.67)	<0.001	1.42 (1.23–1.63)	<0.001
	Ischemic heart disease		1.20 (0.98–1.46)	0.076	1.01 (0.76–1.34)	0.942
	Cerebrovascular disease		1.31 (1.14–1.52)	<0.001	1.13 (0.92–1.38)	0.250
	Chronic obstructive lung disease		1.12 (0.97–1.30)	0.131	0.96 (0.77–1.19)	0.713
	Liver disease		2.46 (2.10–2.88)	<0.001	3.21 (2.68–3.83)	<0.001
	Anemia (Hb <10 g dL^−1^)		3.73 (3.49–4.00)	<0.001	4.77 (4.33–5.25)	<0.001
	Cancer		1.83 (1.70–1.97)	<0.001	2.06 (1.88–2.27)	<0.001
	eGFR mL min^−1^ 1.73 m^−2^					
		≥90	1	(<0.001)	1	(<0.001)
		60–90	1.04 (0.96–1.13)	0.343	0.77 (0.68–0.88)	<0.001
		30–60	2.21 (2.01–2.44)	<0.001	1.17 (1.02–1.35)	0.029
		<30	5.33 (4.73–6.00)	<0.001	3.69 (3.24–4.22)	<0.001
Admission through ED			1.51 (1.41–1.61)	<0.001	1.91 (1.73–2.10)	<0.001
Postoperative admission			0.86 (0.81, 0.92)	<0.001	0.68 (0.62, 0.74)	<0.001
Fluid administration for 72 h						
	NaCl 0.9%, per 100 ml increase		1.00 (1.00, 1.00)	0.018	1.01 (1.00, 1.01)	0.001
	Balanced crystalloid, per 100 mL		0.99 (0.99, 1.00)	0.014	0.98 (0.97, 0.98)	<0.001
	Hydroxyethyl starch, per 100 mL		0.98 (0.97, 0.99)	0.002	0.96 (0.95, 0.98)	<0.001
	Transfusion of packed RBC		2.43 (2.28, 2.60)	<0.001	2.34 (2.14, 2.57)	<0.001
The number of measurements of Cl^−^			1.93 (1.86, 2.01)	<0.001	1.63 (1.54, 1.72)	<0.001
Admission department						
	Internal medicine		1	(<0.001)	1	(<0.001)
	Neurologic center		0.23 (0.21–0.26)	<0.001	0.19 (0.16–0.22)	<0.001
	Cardiothoracic surgical department		0.79 (0.73–0.86)	<0.001	0.52 (0.47–0.59)	<0.001
	Other surgical departments		0.77 (0.71–0.85)	<0.001	0.64 (0.57–0.72)	<0.001

AKI, acute kidney injury; ICU, intensive care unit; APACHE, acute physiology and chronic health evaluation; eGFR, estimated glomerular filtration rate; ED, emergency department; Hb, hemoglobin; RBC, red blood cell; RRT, renal replacement therapy.

## References

[1] Waikar S.S., Bonventre J.V. (2009). Creatinine kinetics and the definition of acute kidney injury. J. Am. Soc. Nephrol..

[2] Bellomo R., Kellum J.A., Ronco C. (2012). Acute kidney injury. Lancet.

[3] Lewington A.J., Cerda J., Mehta R.L. (2013). Raising awareness of acute kidney injury: A global perspective of a silent killer. Kidney Int..

[4] Nash K., Hafeez A., Hou S. (2002). Hospital-acquired renal insufficiency. Am. J. Kidney Dis..

[5] Ostermann M., Chang R.W. (2007). Acute kidney injury in the intensive care unit according to rifle. Crit. Care Med..

[6] Rewa O., Bagshaw S.M. (2014). Acute kidney injury-epidemiology, outcomes and economics. Nat. Rev. Nephrol..

[7] Thakar C.V., Christianson A., Freyberg R., Almenoff P., Render M.L. (2009). Incidence and outcomes of acute kidney injury in intensive care units: A veterans administration study. Crit. Care Med..

[8] Macedo E., Mehta R.L. (2015). Preventing acute kidney injury. Crit. Care Clin..

[9] Pfortmueller C.A., Uehlinger D., von Haehling S., Schefold J.C. (2018). Serum chloride levels in critical illness-the hidden story. Intensive Care Med. Exp..

[10] Shao M., Li G., Sarvottam K., Wang S., Thongprayoon C., Dong Y., Gajic O., Kashani K. (2016). Dyschloremia is a risk factor for the development of acute kidney injury in critically ill patients. PLoS ONE.

[11] Toyonaga Y., Kikura M. (2017). Hyperchloremic acidosis is associated with acute kidney injury after abdominal surgery. Nephrology.

[12] Yunos N.M., Bellomo R., Glassford N., Sutcliffe H., Lam Q., Bailey M. (2015). Chloride-liberal vs. Chloride-restrictive intravenous fluid administration and acute kidney injury: An extended analysis. Intensive Care Med..

[13] De Vasconcellos K., Skinner D.L. (2018). Hyperchloraemia is associated with acute kidney injury and mortality in the critically ill: A retrospective observational study in a multidisciplinary intensive care unit. J. Crit. Care.

[14] Jaynes M.P., Murphy C.V., Ali N., Krautwater A., Lehman A., Doepker B.A. (2018). Association between chloride content of intravenous fluids and acute kidney injury in critically ill medical patients with sepsis. J. Crit. Care.

[15] Sadan O., Singbartl K., Kandiah P.A., Martin K.S., Samuels O.B. (2017). Hyperchloremia is associated with acute kidney injury in patients with subarachnoid hemorrhage. Crit. Care Med..

[16] Berend K., van Hulsteijn L.H., Gans R.O. (2012). Chloride: The queen of electrolytes?. Eur. J. Intern. Med..

[17] Yunos N.M., Bellomo R., Story D., Kellum J. (2010). Bench-to-bedside review: Chloride in critical illness. Crit. Care.

[18] Oh T.K., Song I.-A., Kim S.J., Lim S.Y., Do S.-H., Hwang J.-W., Kim J., Jeon Y.-T. (2018). Hyperchloremia and postoperative acute kidney injury: A retrospective analysis of data from the surgical intensive care unit. Crit. Care.

[19] Hallan S., Asberg A., Lindberg M., Johnsen H. (2004). Validation of the modification of diet in renal disease formula for estimating gfr with special emphasis on calibration of the serum creatinine assay. Am. J. Kidney Dis..

[20] Kellum J.A., Lameire N., Group K.A.G.W. (2013). Diagnosis, evaluation, and management of acute kidney injury: A kdigo summary (part 1). Crit. Care.

[21] Chawla L.S., Eggers P.W., Star R.A., Kimmel P.L. (2014). Acute kidney injury and chronic kidney disease as interconnected syndromes. N. Engl. J. Med..

[22] Armstrong R.A. (2014). When to use the bonferroni correction. Ophthalmic Physiol. Opt..

[23] Murty M.S., Sharma U.K., Pandey V.B., Kankare S.B. (2013). Serum cystatin c as a marker of renal function in detection of early acute kidney injury. Indian J. Nephrol..

[24] Nagami G.T. (2016). Hyperchloremia—Why and how. Nefrologia.

[25] Bank N., Better O.S. (1991). Acid-base balance and acute renal failure. Miner Electrolyte Metab..

[26] Tani M., Morimatsu H., Takatsu F., Morita K. (2012). The incidence and prognostic value of hypochloremia in critically ill patients. Sci. World J..

[27] Krajewski M.L., Raghunathan K., Paluszkiewicz S.M., Schermer C.R., Shaw A.D. (2015). Meta-analysis of high- versus low-chloride content in perioperative and critical care fluid resuscitation. Br. J. Surg..

[28] Kawano-Dourado L., Zampieri F.G., Azevedo L.C.P., Correa T.D., Figueiro M., Semler M.W., Kellum J.A., Cavalcanti A.B. (2018). Low- versus high-chloride content intravenous solutions for critically ill and perioperative adult patients: A systematic review and meta-analysis. Anesth. Analg..

[29] Chappell D., Jacob M., Hofmann-Kiefer K., Conzen P., Rehm M. (2008). A rational approach to perioperative fluid management. Anesthesiology.

[30] Bagshaw S.M., George C., Bellomo R., Committee A.D.M. (2007). Changes in the incidence and outcome for early acute kidney injury in a cohort of australian intensive care units. Crit. Care.

[31] Kellum J.A., Sileanu F.E., Murugan R., Lucko N., Shaw A.D., Clermont G. (2015). Classifying aki by urine output versus serum creatinine level. J. Am. Soc. Nephrol..

[32] Vaidya V.S., Ferguson M.A., Bonventre J.V. (2008). Biomarkers of acute kidney injury. Annu. Rev. Pharmacol. Toxicol..

